# De-implementation of low value castration for men with prostate cancer: protocol for a theory-based, mixed methods approach to minimizing low value androgen deprivation therapy (DeADT)

**DOI:** 10.1186/s13012-018-0833-7

**Published:** 2018-11-29

**Authors:** Ted A. Skolarus, Sarah T. Hawley, Daniela A. Wittmann, Jane Forman, Tabitha Metreger, Jordan B. Sparks, Kevin Zhu, Megan E. V. Caram, Brent K. Hollenbeck, Danil V. Makarov, John T. Leppert, Jeremy B. Shelton, Vahakn Shahinian, Sriram Srinivasaraghavan, Anne E. Sales

**Affiliations:** 1 VA HSR&D Center for Clinical Management Research, VA Ann Arbor Healthcare System, 2215 Fuller Road, Ann Arbor, MI 48105 USA; 2 Department of Urology, Dow Division of Health Services Research, University of Michigan Medical School, 1500 E Medical Center Dr, Ann Arbor, MI 48109 USA; 3 Department of Internal Medicine, Division of Hematology/Oncology, University of Michigan Medical School, Ann Arbor, MI USA; 4 Department of Learning Health Sciences, University of Michigan Medical School, Ann Arbor, MI USA; 5 Departments of Urology and Population Health, NYU Langone Medical Center, New York City, NY USA; 6 VA New York Harbor Healthcare System, 423 E. 23rd St, New York City, NY 10010 USA; 7 Department of Urology, Stanford University School of Medicine, Grant Building, S-287, 300 Pasteur Drive, Stanford, CA 94305 USA; 8 VA Palo Alto Healthcare System, Palo Alto, CA USA; 9 VA Greater Los Angeles Healthcare System, 11301 Wilshire Blvd, Los Angeles, CA 90073 USA; 10 Division of Nephrology, University of Michigan Medical School, Medical School, 1500 E. Medical Center Dr, Ann Arbor, MI 48109 USA; 11 University of Michigan Ross School of Business, 701 Tappan Street, Ann Arbor, MI 48109 USA

**Keywords:** Castration, De-implementation, Choosing wisely, Low value care, Implementation science, Intervention, Formulary restriction, Decision-making, Behavior change, Discrete choice, Stakeholder, Androgen deprivation therapy (ADT)

## Abstract

**Background:**

Men with prostate cancer are often castrated with long-acting injectable drugs termed androgen deprivation therapy (ADT). Although many benefit, ADT is also used in patients with little or nothing to gain. The best ways to stop this practice are unknown, and range from blunt pharmacy restrictions to informed decision-making. This study will refine and pilot two different de-implementation strategies for reducing ADT use among those unlikely to benefit in preparation for a comparative effectiveness trial.

**Methods/design:**

This innovative mixed methods research program has three aims. Aim 1: To assess preferences and barriers for de-implementation of chemical castration in prostate cancer. Guided by the theoretical domains framework (TDF), urologists and patients from facilities with the highest and lowest castration rates across the VA will be interviewed to identify key preferences and de-implementation barriers for reducing castration as prostate cancer treatment. This qualitative work will inform Aim 2 while gathering rich information for two proposed pilot intervention strategies. Aim 2: To use a discrete choice experiment (DCE), a novel barrier prioritization approach, for de-implementation strategy tailoring. The investigators will conduct national surveys of urologists to prioritize key barriers identified in Aim 1 for stopping incident castration as localized prostate cancer treatment using a DCE experiment design. These quantitative results will identify the most important barriers to be addressed through tailoring of two pilot de-implementation strategies in preparation for Aim 3 piloting. Aim 3: To pilot two tailored de-implementation strategies to reduce castration as localized prostate cancer treatment. Building on findings from Aims 1 and 2, two de-implementation strategies will be piloted. One strategy will focus on formulary restriction at the organizational level and the other on physician/patient informed decision-making at different facilities. Outcomes will include acceptability, feasibility, and scalability in preparation for an effectiveness trial comparing these two widely varying de-implementation strategies.

**Discussion:**

Our innovative approach to de-implementation strategy development is directly aligned with state-of-the-art complex implementation intervention development and implementation science. This work will broadly advance de-implementation science for low value cancer care, and foster participation in our de-implementation evaluation trial by addressing barriers, facilitators, and concerns through pilot tailoring.

**Trial registration:**

ClinicalTrials.gov Identifier: NCT03579680, First Posted July 6, 2018.

## Background

Prostate cancer is the most common male cancer in the USA [1]. One in three men with prostate cancer is chemically castrated at some point with long-acting injectable drugs (i.e., androgen deprivation therapy or ADT) [2]. This impacts the well-being of thousands of men annually. Although some patients with prostate cancer benefit from ADT in terms of survival and symptom improvement, chemical castration is also commonly performed when there is no high level evidence for use and little to no health benefits to patients raising questions of low value care. A growing awareness of iatrogenic harms (e.g., heart attack, osteoporosis, diabetes, loss of sexual function) [3], coupled with a lack of evidence supporting chemical castration in many cases [1, 4–6], creates patient safety concerns. Despite this, chemical castration with ADT in low value cases persists as in the case of localized prostate cancer treatment [7].

Because prostate cancer cells are dependent on androgens, e.g., testosterone, androgen deprivation with castration can improve clinical outcomes, for some patients [8]. The highest levels of evidence for chemical castration with ADT injections to treat prostate cancer occur in two scenarios: (1) high risk localized disease in combination with radiation therapy, and (2) metastatic cancer with spread to bones or other organs causing symptoms such as pain [8–11]. However, a significant amount of castration among patients receiving fee-for-service or integrated health system care occurs outside those instances where high levels of benefit exist [1, 4–7]. Neither long-term studies nor current guidelines support castration as primary treatment for localized prostate cancer [11]. For example, using castration for the primary treatment of localized prostate cancer does not improve prostate cancer-specific survival and can be harmful [12], yet remains common in the US Veterans Health Administration [13], a large publicly funded integrated health system, as well as among patients in the US Medicare system [14]. Many times, this castration, which is potentially reversible, is continued indefinitely without clear indication. Even in cases of metastatic prostate cancer without symptoms, an American Society of Clinical Oncology Panel could not recommend initiating treatment with ADT before symptoms of disease progression (e.g., bone pain) occur due to the lack of evidence of an overall survival advantage for early treatment [15].

There are several possible reasons why low value ADT in the treatment of localized prostate cancer persists. First, ADT decreases the serum PSA level, a biomarker of prostate cancer activity, providing false reassurance of a “remission.” This is potentially harmful in a couple of ways. Depriving prostate cancer cells of testosterone too early in the disease process may foster castration resistance, limiting ADT’s effectiveness when it may actually be needed later (e.g., metastatic setting) [16, 17], and PSA is a poor surrogate marker for survival in localized disease so a lowered PSA in this setting creates false optimism [18]. Second, surgical specialists are prescribing a drug with potentially devastating metabolic and cardiovascular effects creating a disconnect between treating PSA levels among men with localized disease and the consequences. Primary care providers are often left to deal with this combination of treating PSA levels without good evidence, and the significant negative health consequences for the patient [19–22].

Ineffective and harmful practices such as chemical castration of patients with prostate cancer outside of the evidence base are ripe for de-implementation. De-implementation, or stopping low value practices, has the potential to improve patient outcomes and decrease healthcare costs [23–26]. For example, stopping chemical castration for localized prostate cancer treatment could prevent harm, and limit spending without affecting survival. However, provider and patient preferences regarding de-implementation are not well understood, and possible de-implementation interventions range from blunt policies to more informed decision-making, potentially with decision support tools to facilitate unlearning [23, 27–29]. Blunt policy interventions such as formulary restriction of ADT (e.g., pre-authorization, order templates) might seem warranted given patient safety concerns, yet could result in significant provider resistance, work-arounds, and even a chilling effect on evidence-supported use of ADT if introduced poorly [30–43]. More nuanced, patient-centered interventions such as informed decision-making (e.g., decision aid, values elicitation) likely involve extra clinical time [44–46]. Both intervention strategies need tailoring for acceptability and feasibility in clinical practice, including piloting prior to trialing [47]. The messaging and operation of strategies to stop low value cancer care hinges on stakeholder input. As many medical practices lack evidence and cause harm, robust behavioral theory-based methods for incorporating provider preferences into de-implementation strategy development will advance both implementation research and practice. While lowering PSA might make sense on the surface, understanding beliefs and preferences for using ADT is a critical step in stopping its low value use. In many respects, this is an ideal model for understanding de-implementation of low value cancer care.

Our preliminary data indicate thousands of men are at risk of ongoing low value castration, especially those with localized disease. Indeed, this calls for effective de-implementation strategies grounded in an understanding of context, provider and patient preferences, and evidence-based behavior change techniques to overcome the wide variation in practice patterns we have observed [48–57]. A significant scientific and clinical knowledge gap remains in prioritizing which barriers to stopping castration in low value settings need to be targeted for effective de-implementation. While a major focus in this study pertains to identifying, prioritizing, and overcoming barriers, the facilitators for stopping ADT which may be transferable across settings also need to be considered [58]. Using a discrete choice experiment (DCE) will allow prioritization of both positive (facilitators, preferences) and negative factors (barriers) to guide theory-based de-implementation strategies as a promising stakeholder-based approach applicable to other low value cancer care [59–63].

## Methods/design

For these reasons, we will examine urologist and patient perspectives on chemical castration with ADT in low value conditions, i.e., as localized prostate cancer treatment. Using a theory-based, exploratory-sequential mixed methods approach to tailor different de-implementation strategies, we will pilot interventions to prepare for a pragmatic, randomized comparative effectiveness trial of two different approaches that vary widely in delivery, impact, and expected results for reducing low value ADT use. We have three specific aims:

- Aim 1: To assess preferences and barriers for de-implementation of chemical castration in prostate cancer**.** Guided by the theoretical domains framework (TDF) [49, 64, 65], we will interview urologists and patients from facilities with the highest, and a few of the lowest, castration rates across an integrated delivery system to identify key preferences and de-implementation barriers, as well as facilitators, for reducing castration as prostate cancer treatment. This qualitative work will inform Aim 2 and gather rich information for our proposed pilot intervention strategies.
- Aim 2: To use a discrete choice experiment, a novel barrier prioritization approach, for de-implementation strategy tailoring. We will conduct a national survey of urologists to prioritize the key barriers identified in Aim 1 for not recommending castration as localized prostate cancer treatment using a DCE. These quantitative results will identify the most important barriers to be addressed through tailoring of our two pilot de-implementation strategies in preparation for Aim 3 piloting.
- Aim 3: To pilot two tailored de-implementation strategies to reduce castration as localized prostate cancer treatment. Building on findings from Aims 1 and 2, we will refine two pilot de-implementation strategies. One strategy will focus on formulary restriction at the organizational level, and the other on decision-making at the physician/patient level. Outcomes will include acceptability, feasibility, and scalability [66] in preparation for an effectiveness trial comparing these two widely varying de-implementation strategies across the integrated delivery system.

### Conceptual framework

**Fig. 1:**
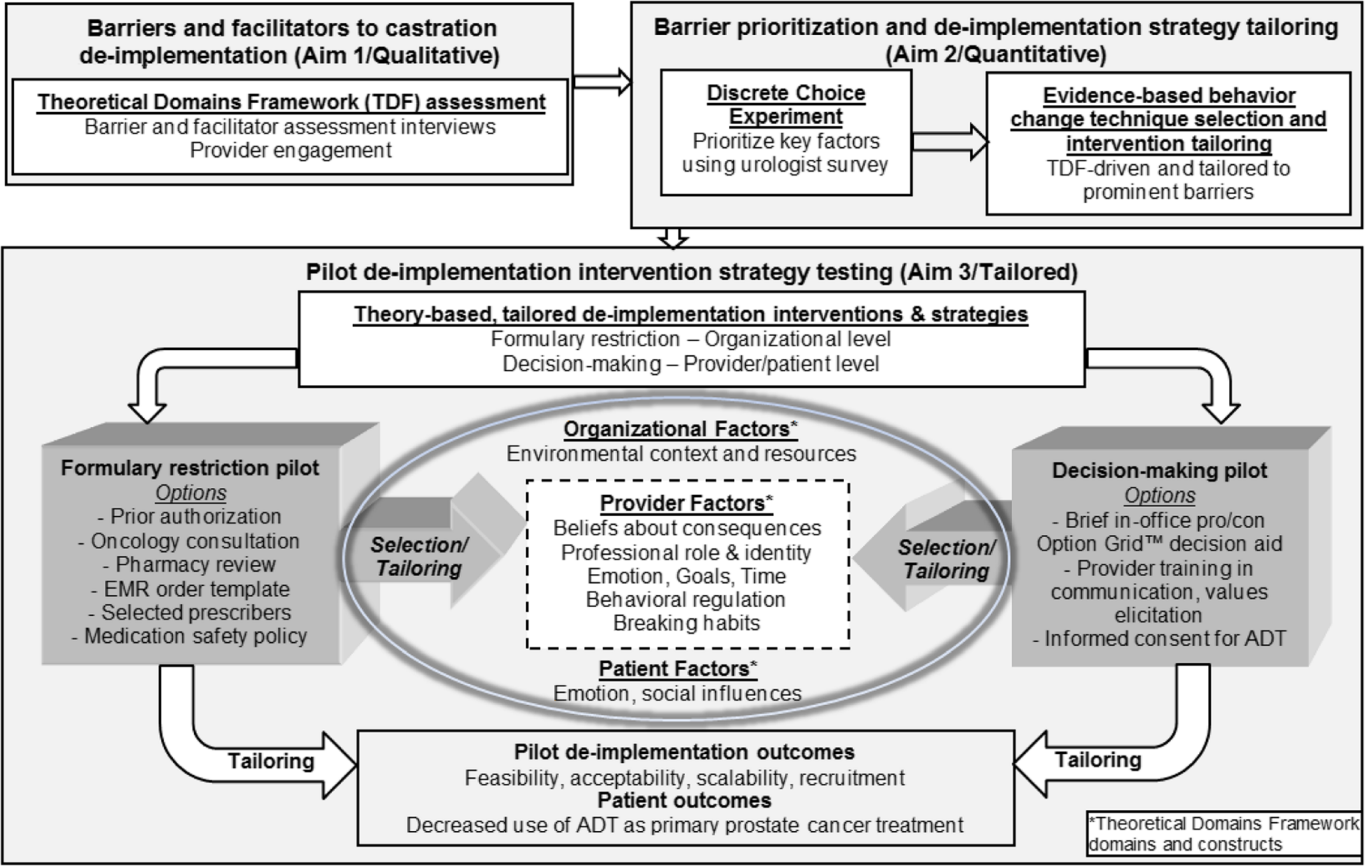
Conceptual model for de-implementation of low value prostate cancer care

It is useful to consider a conceptual model for theory-based qualitative barrier assessment (Aim 1), quantitative prioritization (Aim 2), and piloting of de-implementation strategies tailored to provider behavior change techniques (Aim 3). As illustrated in Fig. 1, we highlight several TDF domains and constructs in our conceptual model that may contribute to organizational, provider, and patient behavior in the setting of ADT for localized prostate cancer. In addition, our qualitative approach allows for flexibility as we conceptualize the main issues when it comes to chemical castration. Last, the quantitative discrete choice methods (Aim 2) create significant opportunities to examine interactions among domains and constructs allowing us to select, tailor, and pilot the most informed organizational and individual level de-implementation interventions during Aim 3.

### Setting

This study is being conducted across several sites in the Veterans Health Administration with the majority of study activities at two sites: the VA Health Services Research & Development Center for Clinical Management Research at the VA Ann Arbor Healthcare System and the Rogel Cancer Center at the University of Michigan.

### Study population and eligibility criteria

We will identify potential study participants using methods successfully used in prior studies of VA providers and prostate cancer survivors [67, 68]. Specifically for the Aim 1 semi-structured provider interviews, we will purposefully sample 10–12 urologists from our integrated delivery system facilities with the highest use of castration as primary treatment to achieve thematic saturation. Likewise, we will also sample 2–4 urologists from facilities with the lowest use of castration. Any urologist who has experience caring for prostate cancer patients on ADT and expresses interest in prostate cancer care will be eligible to participate in semi-structured interviews. We will consider interviewing non-physician providers (e.g., nurse practitioners) if they prescribe a significant amount of ADT. To better understand patient perspectives on not initiating or stopping castration with ADT, we also plan to conduct a limited number of patient interviews (~ 6) from high outlier sites.

For the Aim 2 DCE, we will distribute invitations to the national VA Urology listserv which includes approximately 250 VA urologists. We anticipate this survey will be available over the intranet, as an online survey, as well as available in paper and pen form if preferred. We will use a modified Dillman technique to enhance response rates similar to prior successful survey research [69]. Our engagement with the national VA Urology listserv and the American Urological Association Annual Meeting, alongside operational support from the VA Urology Surgery Advisory Board, should ensure that at least the minimum number of providers needed participate. If needed, we can pursue other organizations (e.g., American Urological Association, Society of Urologic Oncology) given our prominent urologic oncology team.

For Aim 3, we will conduct pilot testing at two sites during years 3 and 4 of this proposal. This will take place at the VA Ann Arbor Healthcare System and University of Michigan’s Rogel Cancer Center Urologic Oncology clinics. The number of patients initiating primary ADT at each site should be adequate for piloting based on preliminary data. However, the primary purpose of the pilots is to assess for issues described in more detail below [70], not effectiveness, so the number of patients on ADT is not critical to success for pilot outcomes.

### Potential human subjects risks and protections against them

This proposal received Institutional Review Board approval from the VA Ann Arbor Healthcare System and the University of Michigan. Aim 1 initially involves analyses of retrospective administrative data to identify facilities, their characteristics, and ADT utilization rates. Study data will come from the VA Corporate Data Warehouse and its Oncology database. This is a centrally maintained national electronic data repository consisting of cancer registry, laboratory, pharmacy, utilization, and administrative data for all patients receiving care in the delivery system. Reported data will be aggregated to the facility level which will not permit identification of individual patients or providers. Next, we will conduct semi-structured interviews with providers and a limited number of patients from facilities with the highest ADT treatment rates for localized prostate cancer. We will audiotape and analyze this data in Aim 1. The risks of the proposed semi-structured interviews are limited, but do include psychological stress related to discussions of appropriateness of prostate cancer treatment with ADT and possible tensions given the low levels of evidence supporting ADT use in localized disease. Another risk is the potential breach of confidentiality of this interview data. Precautions will be taken to protect against any such breach, making the risk low, following standard operating procedures at our Center. Potential human subjects’ risks for the national survey in Aim 2 are low including an email and minimal disruption to provider work flow. With the exception of some demographic information (e.g., age in 5-year increments), the participant information and responses will be kept anonymous. Aim 3 risks for pilot site providers include disruption of clinical workflow and a low likelihood of psychological distress. These risks will be site- and intervention-dependent with a research assistant on-site to assist with recruitment, acceptability of the intervention, feasibility in clinical practice, and data collection and outcome assessment issues. The right of subjects to discontinue their involvement in the research at any time will also be fully disclosed during the informed consent process.

### Participant recruitment and consent

For the qualitative portions of Aim 1, in person and telephone interviews will be piloted with providers at the VA Ann Arbor Healthcare System followed by 12–16 urologists from several other VA Medical Centers as described. Medical Center Directors and Chief Urologists will be contacted via email and brief telephone calls initially and will also be provided information on the nature and purpose of the study, as well as the types of questions their providers will be asked. With approval, urologists who express an interest in participation will be consented by a trained research assistant, who will provide them with information on the nature and purpose of the study, as well as the types of questions that they will be asked (focused on ADT treatment for prostate cancer). Written or recorded phone consent will be obtained. Those who are eligible will then be called at a later date for a formal interview lasting approximately 45 min. Interviews will be conducted by the study team and will be audiotaped. We will use similar recruitment methods for our limited number of patient interviews.

For the survey portion of Aim 2, providers will be identified through a national urologist email listserv and contacted/recruited via email to participate in a DCE survey. An informed consent form will be included, highlighting the nature and purpose of the proposed research as well as sample questions. Participants will be asked to electronically sign the consent form, and will be clearly offered the option of not participating.

Urologists at the pilot sites in Aim 3 will be given an overview of the site-specific intervention, highlighting the nature and purpose of the proposed research, as well as what they should expect to encounter during routine clinical care should they agree to proceed with the study. Pilot site urologists will be asked to sign the consent form by the site research assistant, if they agree to participate, and will be clearly offered the option of not participating.

### Aim 1 methods

The goal of Aim 1 is to clarify barriers and facilitators to stopping castration with ADT as primary prostate cancer treatment using an individual behavior change framework, the theoretical domains framework (TDF) [71]. This approach will identify key barriers to de-implementation of low value ADT-based castration. We will conduct semi-structured interviews with urologists to clarify preferences for (i.e., facilitators) and barriers to stopping ADT use. This will prepare us for development of a theory-based DCE among a national sample of urologists in Aim 2 to quantify the relative importance of barriers, and to direct intervention strategy tailoring to increase acceptability and effectiveness. Embedding use of the TDF within a DCE is particularly innovative.

#### Semi-structured provider and patient interviews

In order to maximize the likelihood that data collected from participants will address our study aims, we will pilot interviews with consultants and providers on our study team. Using the TDF to understand provider and patient ADT behavior will help (1) map our findings to key TDF behavioral constructs; (2) select appropriate evidence-based behavior change techniques (BCTs) [72, 73]; (3) understand barriers, facilitators, and stated preferences for our de-implementation interventions; and (4) advance implementation science. We will explore provider and patient (1) understanding of ADT as primary prostate cancer treatment, (2) harms of castration, (3) behavioral determinants and barriers to delivering (receiving for patients) ADT as primary prostate cancer treatment for localized prostate cancer, and (4) intention to treat localized prostate cancer with ADT.

Interviews will be audiotaped and transcribed verbatim for content. We will use a preliminary coding scheme based on the TDF and prior work [52, 74], consisting of a three step process including (1) coding affirmative and negative utterances regarding ADT use as localized treatment into TDF domains, (2) collecting responses across respondents into themes (e.g., “ADT prevents cancer spread” into a “ADT is beneficial” theme, “urologists should be able to treat patients as they wish” into a “Physician autonomy” theme), and (3) tallying the total number of mentions per theme, as well as conflicting beliefs within a theme (e.g., ADT is good vs. ADT is bad), according to the TDF domains, with particular emphasis on those included in our conceptual model. The principal investigator and research assistant will both independently review and code at least two transcripts and meet regularly to compare coding results until reaching agreement on code definitions and establish the reliability of the coding process (> 80% simple agreement). During this process, the research team will also meet to categorize the codes into TDF domains, identify emerging themes, and document ongoing data interpretation in memos. The research assistant will code the remaining transcripts. Once data are coded, we will use QSR NVivo™ software to organize the data. The research team will meet regularly to discuss code summaries and memos, developing findings with a focus on informing the Aim 2 discrete choice tool [75]. At the end of this aim, we will have identified the highest frequency themes and themes with conflicting provider and patient beliefs across TDF domains for de-implementing ADT.

### Aim 2 methods

The goal of Aim 2 is to then prioritize barriers and facilitators to de-implementation of chemical castration with ADT discovered in Aim 1. The highest priority barriers will need to be addressed during strategy development and tailoring for our pilot interventions in Aim 3 to support acceptability and feasibility in practice. We will accomplish this using a DCE. This stated preference method drives marketing strategy development based on stakeholder preferences for a given practice and is a promising applied approach for health care optimization [59–62, 76, 77]. Real-world DCE examples include prioritizing provider preferences related to hospital consultant work and electronic medical record use [78, 79].

In our DCE, the barriers and themes with the highest frequency and most conflicting beliefs across respondents identified in Aim 1 will be refined and presented as the “attributes” and associated levels will be developed. In short, we will use data obtained from Aim 1 to develop TDF-based choice sets for inclusion in a national urologist DCE. Once we have the most important, not just most common or conflicting, themes and barriers based on a national urologist DCE, we can select the most effective evidence-based behavior change techniques to direct de-implementation tailoring efforts in Aim 3.

In a DCE, stakeholders, in this case urologists prescribing ADT, choose between hypothetical alternatives described by themes and barrier characteristics identified from Aim 1 (i.e., attributes and levels). The five or six highest frequency and most conflicting themes based on Aim 1 findings will be varied across hypothetical examples. Through systematically varying a set of levels in a series of choice sets where providers are asked which option they most prefer, we gather critical data on castration preferences and tailoring for our pilot interventions. This results in a preference structure where certain attributes (barriers) are most important across the respondent sample. The data obtained on the levels of attributes shows which direction the attribute is most favored. In an example of whether high or low physician autonomy or level of evidence is driving ADT treatment decisions, for each choice set given, we will ask respondents to choose the situation that they would most prefer when prescribing ADT for their prostate cancer patients (choice A, choice B, or neither). We may ask participants: “Please select the situation that you most prefer when prescribing ADT as primary treatment.” Candidate themes, attributes, and levels include, for example, the ability to make the final recommendation (levels: yes, no), and the amount of clinical time required (levels: 15 min, 30 min). We will use an opt-out option to ensure realistic scenarios. Data from this DCE survey will identify leading themes and attributes (barriers).

#### Designing and fielding the national urologist DCE

We will decide on the number of attributes based on Aim 1 findings, likely 4–6 per scenario as suggested by the literature [63]. The DCE will measure TDF-based themes across 10–20 choice sets, pared down using a fractional factorial approach, to a reasonable number of hypothetical scenarios per respondent to minimize respondent burden. We will use software to construct the choice sets to identify the least number of choice sets completed by the least number of respondents to detect significant differences among covariates in our model. We will draft a DCE survey tool for refinement and pre-testing on our study team and a sample of urologists. We will modify it accordingly based on pre-testing. We will then distribute invitations to participate to the national VA Urology listserv which includes approximately 250 VA urologists. As described above, we may include other organizations if our response rate is lower than expected.

#### DCE statistical analysis

Discrete choice experiments are based on random utility theory which assumes that participants will select responses with the most personal utility [78]. Because respondents respond to a variety of choice sets, we will be able to estimate the relative priority of our barrier attributes and their levels. We will model urologists’ stated preferences providing quantitative information about the relative value, or utility, providers place on barrier attributes such as physician autonomy or clinical time, for example, using the equations below. If 50% of the 250 urologists respond: 125 surveys with ~ 5 scenarios − 625 scenarios × 5 attributes = 3125 data elements for analysis. Our methods will adjust for dependency of responses within individuals (autoregression) as they respond to different choice sets and will be modeled after published DCEs according to the following example equation:

$$ \mathrm{Utility}=\left(\mathrm{constant}\right)+\upbeta 1\left(\mathrm{e}.\mathrm{g}.,\mathrm{physician}\ \mathrm{autonomy}\right)+\upbeta 2\ \left(\mathrm{e}.\mathrm{g}.,\mathrm{clinical}\ \mathrm{time}\right)\dots $$

We will assess for model fit and need for random parameters among the attributes. Attribute levels will range from − 1 for our reference level to + 1 for the alternative to allow determination of relative importance. Our outcomes will be based on the beta parameter values (β1, β2, etc.) and standard errors that correspond to each attribute where a negative value will indicate preference for the reference group, statistical significance will be set at 0.05. Once we have our leading barrier attributes and corresponding TDF domains, we will select the most relevant candidate evidence-based behavior change technique components based on prior work by Michie et al. [49, 73] to guide tailoring of pilot de-implementation interventions. We also plan to adjust our models for facility-level ADT rates, and perform a subgroup analysis for facilities with high primary ADT rates to better understand barriers to tailor toward (Table 1).

**Table 1:** Data sources and variables

Information	Source	Variables
Aim 1		
Barriers and facilitators to stopping low value chemical castration	12–16 urologists, selected patients from high and a few low ADT facilities	Semi-structured interview data coded into themes and TDF domains for use in Aim 2
Aim 2		
Themes, barriers, attributes for discrete choice scenarios	TDF domains and themes related to treating localized prostate cancer with ADT from Aim 1	4–6 attributes with varying levels (e.g., *physician autonomy*, *clinical time*)
Discrete choice experiment (DCE)	Mixed multinomial logit analysis of DCE survey results among national sample of urologists	Choice sets, attributes, model outcomes (barrier attribute weights)
Behavior change techniques for most relevant attributes for Aim 3	Michie et al. *The Behavior Change Wheel: A Guide to Designing Interventions* [72]	Relevant TDF domains from the DCE model output for use in tailoring strategies

### Broad intervention strategies require tailored design: formulary restriction and decision-making

There are several potential implementation strategies to de-implement low value castration within the broad categories we identified for this proposal. We focus on formulary restriction and informed decision-making because of their difference in key attributes, including level (organizational vs. individual), likelihood of quick success vs. long-term sustainment, and effort required by clinicians. We describe some possible strategy design features briefly for each type in Table 2. While several options exist, there is no existing evidence to inform the best approach from the provider perspective. Aims 1 and 2 will inform which of these strategies is likely to be most acceptable to clinicians, and provide data needed to tailor them. For example, we do not know how a blunt formulary restriction intervention would be received by providers considering primary ADT treatment.

**Table 2:** Examples of potential pilot de-implementation interventions

Formulary restriction	
Prior authorization - Oncology consultation - Pharmacy review	Used in infectious disease
Criteria for use - EMR order template - Selected prescribers	Currently used for restricted drugs
Medication safety (VAMedSAFE)	Evaluate, educate, and prevent adverse events
Decision-making	
Decision aid using a brief in-office pro/con (e.g., Option Grid™)	Commercialized shared decision-making for prostate cancer
Provider training in communication and values elicitation	Evidence-based practice though difficult to implement/sustain
Informed consent for ADT	VA iMed consent

While formulary restriction of ADT for localized prostate cancer seems warranted, we may find that it is widely considered unacceptable to providers and patients. Nor do we know how shared or informed decision-making can be efficiently operationalized in a clinical setting for patients considering castration for localized disease. By tailoring each strategy using behavior change techniques and barrier solutions derived from Aims 1 and 2, we believe we can design implementation interventions that will be accepted by providers, but still allow us to test differences in the widely varying mechanisms of action. We will refine these approaches through robust efforts and finding from Aims 1 and 2 (Table 1) and the expertise of our trans-disciplinary investigative team.

### Aim 3 methods

Based on findings from Aims 1 and 2, Aim 3 pilot work plays a critical role to help us understand the acceptability, feasibility, and scalability of these complex interventions in preparation for a full-scale randomized de-implementation evaluation trial. In fact, the UK Medical Research Council guidance indicates piloting is essential to complex intervention development and testing prior to large-scale evaluation [47]. The main goal of both pilots will be to decrease castration rates for patients with localized prostate cancer, in a way that is acceptable to patients and to the clinicians who treat these patients. We are purposely choosing intervention strategies from opposite ends of the behavior change continuum because of their evidence-based potential to change provider behavior. Specifically, we are selecting one approach (formulary restriction policy) that operates at the organizational level and is widely perceived as a forcing function, giving providers little leeway to exercise judgment. The other, physician/patient shared or informed decision-making, operates at an individual and dyadic level, and is perceived as maximizing the opportunity for discussions between patients and providers. The first approach requires little to no learning on the part of providers, while the second requires considerable upfront learning (“cost” to the provider and possibly also to the patient). This approach sets up a testable hypothesis for our subsequent comparative effectiveness trial, that a blunt de-implementation policy may be effective in the short term but that it will lose its effects as providers learn work-arounds. Conversely, a shared or informed decision-making approach to de-implementation might take longer to observe measurable decreases in castration rates, but its effects will create sustainable change as providers internalize and routinize this clinical practice.

#### Methodological issues to be addressed in de-implementation pilots

A well-designed pilot study has many purposes, including testing methods of recruitment, selecting the most appropriate primary outcome, testing acceptability of the intervention by stakeholders, ironing out feasibility and fidelity issues, developing the full study protocol, and estimating sample size for a full trial [70]. As highlighted in implementation literature, preparation and planning are central to successful pilot development and implementation [47, 58, 80]. The need for clear outcomes (e.g., inappropriate castration rate), systematic, theory-based interventions to change provider behavior (i.e., TDF-driven), and a timetable are necessary to successfully set up our full-scale evaluation trial. Although it is likely that chemical castration rates among prostate cancer patients will be our primary outcome for the full evaluation trial (# of primary ADT patients with localized prostate cancer/total incident prostate cancer patients), we will also need to consider implementation outcomes (e.g., feasibility) and hybrid study designs. Further refinement in the pilot studies will allow us to explore other outcomes including the total number of ADT injections as we will also be working to stop treatment among those with localized disease who have been continued on ADT. As illustrated in Table 3, the piloting of the intervention strategies will focus on four major methodological issues. We will examine issues surrounding recruitment, acceptability, feasibility, scalability, and data collection for the full-scale trial [66].

**Table 3:** Methodological issues requiring Aim 3 pilot evaluation prior to a full-scale de-implementation trial

Issue	Assessment	Potential outcome
Recruitment randomization scalability	Monitor proposed recruitment strategy at each facility; check practicality of cluster randomization of facilities; identify issues of participation refusal or withdrawal; acceptability of randomization; number of eligible participants per month; compare clinic flow across recruitment strategies	Select most effective recruitment and randomization strategy; trial messaging to sites; discern patient, provider and cluster sample sizes; refining eligibility screening
Acceptability of intervention	Check acceptability of interventions with urologists and clinic staff at pilot sites; settings for each intervention; consent and documentation practices; tailoring strategies are acceptable; timing of intervention relative to visit	Identify acceptable components of each intervention in clinical practice; consent processes; efficient documentation practices
Feasibility in clinical practice	Assess burden on clinic staff and providers to participate; monitor clinical time and workflow; assess adherence to intervention; technical performance of EMR-based intervention(s); participants representative of those expected in full-scale trial; intervention fidelity	Time and resources needed to roll out in randomized sites; learn research and clinic administrative staff roles for trial; standardization; scheduling practices
Data collection and outcome assessment	Monitor follow up practices for patients on ADT; monitor for asymmetric attrition/retention across intervention sites; missing data; review choice of primary outcome, study design; effect variability	Willingness to participate by intervention preference; effect size; consider hybrid study; duration; full-scale protocol

## Discussion

Many men with prostate cancer are castrated with long-acting injectable drugs. Although some patients benefit, it is also used in patients who have little or nothing to gain, such as men with localized prostate cancer. The best ways to stop, or de-implement, low value cancer care are unknown. A significant scientific and clinical knowledge gap remains in prioritizing which barriers to stopping castration in low value settings need to be targeted for effective de-implementation. Using a mixed methods approach, we will identify, refine, and pilot two different approaches for reducing low value ADT use in preparation for a randomized comparative effectiveness trial. In doing so, this proposal will address important issues surrounding provider behavior change and serve as a model to decrease overtreatment more broadly. Throughout this project, we will keep a broad focus so that our work lays a foundation for transforming how and why castration is performed for prostate cancer treatment. This work will advance de-implementation science for low value care and foster participation in our subsequent de-implementation evaluation trial by addressing preferences and concerns through pilot tailoring.

### Dissemination

While the next steps for this work will be a cluster randomized comparative effectiveness trial, setting up this complex trial will create opportunities for dissemination. We will publish at least one manuscript per research aim in peer-reviewed journals, as well as submit at least one abstract to clinical, quality improvement, and/or implementation research meetings. We will convene a Steering Committee and update this group through quarterly phone calls as we progress through the research plan. In addition, we will present our findings to the American Society of Clinical Oncology, Society of Urologic Oncology, and the Association of VA Hematology/Oncology as an opportunity to include a de-implementation of low value cancer care theme in their annual meeting agendas. We will brief relevant operational partners annually including the VA National Program Director for Oncology and the VA National Urology Surgery Advisory Board. We will also share our findings and recruit for our cluster RCT at the Annual AUA Meeting.

## Trial status

Provider recruitment for Aim 1 started in August 2018.

## Abbreviations

ADT: Androgen deprivation therapy
AUA: American Urological Association
